# Supplementary material for: How do public services supply, livelihood capital, and livelihood strategies affect subjective poverty?
Source: PLoS One. 2023 Oct 10;18(10):e0292651. doi: 10.1371/journal.pone.0292651 (PMC10564167; doi:10.1371/journal.pone.0292651)
Supplement: Attachment — Submitted filename: Response to Reviewers.docx [file pone.0292651.s002.docx]

**Detailed response to reviewers**

Reference: PONE-D-22-12739

Title: How do public services supply, livelihood capital, and livelihood strategies affect subjective poverty?

Journal title: PLOS ONE

Authors: Li Chen, Yuanquan Lu, Yuan Meng

Dear Editors and Reviewers,

Thanks for your letter and comments on our manuscript titled "How do public services supply, livelihood capital, and livelihood strategies affect subjective poverty?" (PONE-D-22-12739). These comments helped us improve our manuscript and provided important guidance for future research.

We have addressed the editor's and the reviewers' comments to the best of our abilities and revised the text to meet the PLOS ONE style requirements. We hope this meets your requirements for a publication.

We marked the revised portions in red in the revised manuscript with track changes. The main comments and our specific responses are detailed below:

Reviewer:

Consider broadening the idea that objective and subjective poverty are not equivalent. Pag.3.

Response:

Thanks to the reviewer' opinions. We have added a part of the content on page 3 of the article related to the different concepts of subjective and objective poverty. The following are specific additions: The objective poverty perspective describes poverty as a lack of ability to maintain a basic state of survival and security, based on two ideas: subsistence and basic needs. The idea of subsistence was first introduced in the book "*Poverty: A Study of Urban Life*" by Rowntree, a British scholar, and has since been adopted by many governments and international agencies, such as the United States, China, and the World Bank. The basic needs approach is no longer limited to material satisfaction such as food, clothing, and shelter to sustain livelihoods but also focuses on social security such as public sanitation, education, and cultural facilities^[4]^. Subjective poverty, the self-perception of poverty, is a self-assessment of people's undesirable lifestyles rather than a determination of others. The concept first emerged as a subjective poverty line in the academic history of scholars' rethinking of the objective poverty line, and foreign researchers, represented by Dutch scholars, pioneered the subjective poverty line measure in the 1970s. Subjective poverty and objective poverty are not equivalent. Koczan defined poverty in two aspects. One is objective economic poverty, where income is inadequate to cover daily expenses; the other is psychologically subjective poverty^[5]^. The subjective evaluation of whether one is in poverty is based on property and income, educational opportunities, pension and medical security, and decision-making participation. In contrast, subjective poverty is more comprehensive. It is an individual's all-around evaluation based on all of their attribute values. It also comprises residents' assessments of the income distribution, educational resource distribution, and social security systems. Subjective poverty additionally includes the overall evaluation of the effect of policy implementation. Having equal rights in income distribution is an important value in China.

Reviewer:

Consider including the analysis techniques used for measurement in quantitative methods.

Response:

Thanks to the reviewer' opinions. All things considered, we think the reviewer's opinion is very good, and we transferred the measurement analysis to data, variables and methods.

Reviewer:

It is recommended to cite the bibliographic references of the studies that justify the idea. Page 3, subtitle 2.1

Response:

Thanks to the reviewer' opinions. We have added the following bibliography in section 2.1. Alem et al. found that families with a history of poverty will continue to think they are in poverty even if their material consumption increases. Mahmood et al. use Pakistan Panel Household Survey 2010 data. They find that the determinants of subjective poverty are not limited to household consumption but include physical health, food security, sanitation facility, household size, household demographic structure, and agriculture land ownership. Other remaining content can be viewed in the revised manuscript.

Reviewer:

It is recommended to check which bibliographic citation is the one that begins the paragraph. Page 4, second paragraph.

Consider the bibliographical references of the studies mentioned on page 6, thirteenth paragraph.

Consider including the bibliographical references of these studies p. 9 start of the paragraph.

Response:

Thanks to the reviewers for their detailed comments. We have revised the content and literature one by one, adding relevant literature and removing some literature in the areas mentioned by the reviewers. The following are some of the modifications, the details of the specific modifications are marked out in red in the revised manuscript with track changes.

According to the existing research results, most of the literature discusses the impact of differences in household micro-features on subjective poverty from multiple aspects, such as population, economy, and policy. Scholars have found that demographic characteristics, including family consumption, household size, marital status, gender differences, educational background, and ancestry, are significantly related to subjective poverty^[2,3,6]^. Nándori analyzes subjective poverty in Hungary and finds that Roma descent, entitlement to social support, and unemployment affect subjective poverty^[7]^. Alem et al. find that families with a history of poverty will continue to think they are in poverty even if their material consumption increases^[8]^. Mahmood et al. use Pakistan Panel Household Survey 2010 data, and they find that the determinants of subjective poverty are not limited to household consumption but include physical health, food security, sanitation facility, household size, household demographic structure, and agriculture land ownership^[3]^.

The economic aspect mainly involves the relationship between subjective poverty and factors such as socio-economic background, income level, household disposable income, and employment status^[9]^. It has been found that all of these factors significantly affect the occurrence of subjective poverty directly or indirectly^[1,3]^. Joyce and Ziliak compare the causes of relative poverty in Britain and the United States and find that relative poverty in Britain is related to working households, while low-skilled is the main cause of relative poverty in the United States^[10]^. Some scholars have also studied the relationship between policy and poverty. They have found that Brazil's rural social assistance pensions have reduced the incidence of poverty among rural elderly by 95%. Canada's social security system reform can also significantly reduce the incidence of poverty. Chile's tax-financed pension measures likewise significantly improve its poverty-alleviation effect^[11]^. Li and Li find that the anti-poverty relocation and settlement program affects subjective well-being by affecting the probability that people will be exposed to risks due to policy^[12]^. González and Villanueva discussed the relationship between non-agricultural activities and employment and wage. The study found that as long as the economic public policy makers take preventive measures in the labor market, the non-agricultural employment index will increase^[13]^.

Reviewer:

Consider the full citation. Pag.5, subchapter 3 first paragraph.

Consider the relevance of the idea of reforms and methods with the dimensions used. Pag.6 third paragraph.

Consider that this idea is already implicit in the previous paragraph. Page 6 line 1, fourth paragraph.

Response:

Thanks to the reviewers for their detailed comments. We are sorry that this part was not clear in the original manuscript. We should explain more clearly. We think that these two sections need further refinement, so we have modified this section. We have modified the original page 5 and 6 (3. Theoretical basis and research hypothesis).

We have adjusted the similarities between the content of the preceding and following paragraphs. We removed duplicate and similar content and added some new content.

We deleted this paragraph:

We based on Koczan’s subjective poverty theory, and establish a theoretical framework in which public services supply, livelihood capital, and livelihood strategies affect subjective poverty. Residents’ psychological and actual poverty or inequality may become obstacles to a national government’s ability to implement a new deal or to carry out reforms. Unreasonable policy formulation or improper implementation after policy formulation may lead to the possibility of poverty, sometimes policies aimed at curbing poverty can even exacerbate the occurrence of poverty. Therefore, a better understanding of people’s subjective poverty and its influencing factors will help a nation’s government to implement more effective public services and to choose the correct order of reforms and communication methods.

We have revised this entire section to read as follows:

The concept of "sustainable livelihoods" originated from the expansion and reflection of anti-poverty research by such scholars as Amartya Sen^[14]^. At the end of the 20th century, Chambers and Conway conceptualized the term "livelihood" for the first time as a general term for individuals' ability to earn a living^[15]^, the assets acquired, the external support that can be used, and the actions centered on income creation for the long-term improvement of production and living conditions. This reveals, to a certain extent, that the core of livelihood is "the action and process of livelihood capital conversion based on the exchange of livelihood capital"^[3]^. Capital conversion aims to promote the sustainability and continual expansion of livelihoods. Abundant livelihood capital can improve the quality of personal production and life, enhance the ability to resist risks, and help block intergenerational poverty transmission. Different types of livelihood capital can also be transformed into each other and thus produce different welfare states.

In his report "Our Common Future," Brundtland first introduced sustainable livelihoods at the 1987 United Nations Conference on Environment and Development. In the 1990s, the concept of sustainable livelihoods evolved after Western scholars gave a clear definition of livelihoods, and the sustainable livelihoods approach was developed by international agencies such as UNDP, CARE, DFID, and FAO as "a method to better capture the complexity of livelihoods, understand the impact of livelihood strategies on poverty, and identify which interventions to adopt." The sustainable livelihoods framework Department for International Development (DFID) proposed is the most widely used in the Sustainable Livelihoods Guide^[16]^. We will use this theory to discuss the choice of the four types of public services supply, livelihood capital, and livelihood strategies, including physical, human, social, and financial, to reveal the impact of individual livelihood systems on subjective poverty^①^.

Reviewer:

Consider expanding the idea that: economic development lacks fundamental internal motivation. To strengthen the foundation of the paragraph. Page 6, eleventh paragraph

Response:

Thanks to the reviewers for their detailed comments. We are sorry that this part was not clear in the original manuscript. I should have explained that we have added this section: with the rapid development of industrialization and urbanization, many high-quality laborers have flowed into big cities. Rural areas or underdeveloped cities only have a handful of low-quality labor. And coupled with the impact of multiple factors such as environment and resources. This leads to economic development lacking fundamental internal motivation. Making some groups vulnerable to subjective poverty.

Reviewer:

What are those non-agricultural media strategies or criteria for measuring this dimension?

Response:

Thanks to the reviewers for their detailed comments. We are sorry that this part was not clear in the original manuscript. We should have explained that we refer here to non-agricultural strategies, other forms of work other than agricultural activities. Such as chefs, welders, cleaners, or other any non-agricultural activities.

Reviewer:

What is the bibliographic reference of the study mentioned in the paragraph? Pag.7 last paragraph.

Response:

Thanks to the reviewers for their detailed comments. This part of our content is a reference to Ogun's article on the subject. We put it in reference 17. Ogun used structural vector autoregressive technology to study the physical infrastructure in Nigeria from 1970 to 2005. They found that large-scale investment in the infrastructure of a region will greatly reduce poverty^[17]^.

17. Ogun T P. Infrastructure and poverty reduction: Implications for urban development in Nigeria. Urban Forum. 2010; 21(3): 249-266.

Reviewer:

Consider using a footnote or expanding your reference in the paragraph to DFID. Page 8, last paragraph.

Response:

Thanks to the reviewers for their detailed comments. We thought this section really needed additional relevant content, so we added something about DFID. Brundtland first introduced sustainable livelihoods in his report "Our Common Future" at the 1987 United Nations Conference on Environment and Development. In the 1990s, the concept of sustainable livelihoods evolved after Western scholars gave a clear definition of livelihoods, and the sustainable livelihoods approach was developed by international agencies such as UNDP, CARE, DFID, and FAO as "a method to better capture the complexity of livelihoods, understand the impact of livelihood strategies on poverty, and identify which interventions to adopt." The most widely used is the sustainable livelihoods framework that Department for International Development (DFID) proposed in the Sustainable Livelihoods Guide.

Reviewer:

Consider mentioning the full names of the 5 scales or value. Pag.14 start of paragraph.

Response:

Thanks to the reviewers for their detailed comments. We are sorry that this part was not clear in the original manuscript. I should have explained that we revised the mentioned five scales to full names, from "up," "middle up," "middle," and "middle down" to "down,"

Reviewer:

Consider that 14 criteria are mentioned to measure the variable and only 2 are mentioned.

Response:

Thanks to the reviewer' opinions. We have refined this section to list all 14 areas. The question mainly includes medical and health services, social security services, protection of the environment, protecting political rights, maintaining social security, integrity and punishment of corruption, fairness in law enforcement, economic development, employment expansion, open government information, service consciousness, safeguarding education equity, safeguarding food and drug safety and overall work and services. Therefore, this article first conducted a reliability analysis.

Reviewer:

It is necessary to carry out a discussion of the results using recent specialized literature. It is also recommended that the implications of the results be noted and discussed.

Response:

Thanks to the reviewer' opinions. We added the most recent literature in the literature review section and analyzed and organized this literature, and selectively removed some of the older literature originally cited in the article. The following are the new literature we have added:

Li Zhao, Jianping Yang. How does Sustainable Livelihood Capital Alleviate Relative Poverty in the Post-poverty Era? *Scientific Journal of Economics and Management Research.* 2021; (3).

Wang M Y, Li M, Jin B, Yao L, Ji H. Does Livelihood Capital Influence the Livelihood Strategy of Herdsmen? Evidence from Western China. *LAND.* 2021; 10 (7).

Yi Xiao, Xixi Tan, Lu Pan. Difference of Farmers' Livelihood Capital before and after Rural Tourism Development. *Computational intelligence and neuroscience.*2022.

Tsaurai K. Human Capital Development, Remittances, and Poverty in Central and Eastern European Countries: What Do the Data Tell Us? *COMPARATIVE ECONOMIC RESEARCH-CENTRAL AND EASTERN EUROPE*. 2022; 25 (1): 23-38.

Li C, Li M L. The Policy Information Gap and Resettlers' Well-Being: Evidence from the Anti-Poverty Relocation and Resettlement Program in China. *INTERNATIONAL JOURNAL OF ENVIRONMENTAL RESEARCH AND PUBLIC HEALTH*. 2020; 17 (8).

Reviewer:

Section 6 must be separated into two; discussion of results and conclusions. This is noted in the document.

Response:

Thanks very much for the reviewer's suggestion. We put the discussion and conclusion together in the original article. The first two paragraphs are the discussion, followed by the conclusion. This may not be clear enough, so we have separated the original Part 6 and made the discussion and conclusions separate chapters. Chapter 6 is the discussion and Chapter 7 is the conclusion.

Editor:

According to reviewer 1, I am suggesting the following references.

Response:

Thanks to the editor for the comments. We read those articles carefully and then selected some articles to add to the literature review in relation to the content of our paper. These articles are very helpful for us to revise our papers.

Thanks to the reviewers and editors for their comments. We have made the following additional changes to the text.

We also revised the abstract, introduction, and figures. We hope it will make the paper look clearer. The specific changes are marked in red in the revised manuscript with the revised manuscript with track changes.

Original abstract:

Poverty governance is regarded as a serious challenge for China; poverty reduction is put in a prior position on the policy agenda. Getting out of poverty is the premise of building a harmonious society and achieving common prosperity. It has been studied intensively in different objective dimensions of Chinese poverty by researchers, and little attention has been paid to subjective poverty.

Modified abstract:

Poverty is not only an economic problem but also a social problem, and there are certain limitations of objective poverty based on the population's income. It does not reflect the residents' true feelings regarding education opportunities, pension and medical security, and participation in decision-making. Researchers have studied it intensively in different objective dimensions of Chinese poverty, and little attention has been paid to subjective poverty.

Original introduction:

Poverty governance is globally regarded as a serious challenge for many developing countries. Poverty reduction is put in a prior position in the policy agenda. The United Nations’ Sustain‑able Development Goals (SDGs) which was passed in 2015 put “No Poverty” as the first goal, specifically to “End poverty in all its forms everywhere” by 2030 (Hanjie Wang et al., 2020). China is no exception. Over the past 40 years, by 2020, China is completed a campaign which is called “The Targeted Poverty Alleviation Policy”, full implementation of the targeted poverty alleviation strategy, the basic logic and operating mode of China's poverty governance have been completely changed. This has offset the decline in the effect of poverty reduction stemming from the decline in economic growth and inequality in income distribution. Thus, long-standing poverty alleviation goal deviation and poor poverty alleviation performance problems have been improved. According to the current standards, 98.99 million rural poor people have been lifted out of poverty. A total of 832 poor counties have been lifted, and 128,000 poor villages have been listed. The overall regional poverty has been resolved. It is well known that there are many dimensions to defines poverty. From a long time, relatively poverty still exists in China. In addition, poverty can not only be measured through the wealth, but also a subjective feeling (Gustafsson & Ding, 2020). Mahamoud et al. (2019) shows the difference between Pakistan's objective poverty and subjective poverty, as they are determined by different factors.

Modified introduction:

Poverty governance is globally regarded as a serious challenge for many developing countries. The United Nations' Sustain‑able Development Goals (SDGs) put "No Poverty" as the first goal, specifically to "End poverty in all its forms everywhere" by 2030^[1]^. China is no exception. Over the past 40 years, China has completed a campaign called "Targeted Poverty Alleviation Strategy." This campaign has enabled China to achieve remarkable poverty governance results and contribute greatly to the world's poverty reduction efforts. This has offset the effect of the decline in economic growth and inequality in income distribution on poverty. According to the current standards, 98.99 million rural poor people have been lifted out of poverty. A total of 832 poor counties have been lifted, and 128,000 poor villages have been listed. Chinese overall regional poverty has been resolved, and it has enhanced people's ability to work. Although the objective poverty problem has been solved, subjective poverty has become the main social problem on China's new journey of building a comprehensive socialist modern state^[2]^. And follow-up plans and measures are also needed to sustain the results of poverty elimination. Mahmood et al. found the difference between Pakistan's objective and subjective poverty, determined by different factors^[3]^.

Thank you again for taking the time to review our manuscript and provide valuable feedback. We greatly appreciate your expertise and insightful comments, which have undoubtedly improved the quality of our research. We also extend our gratitude to the editors for their assistance throughout the review process. Thank you for your support and guidance. We look forward to hearing from you regarding our submission. We would be glad to respond to any further questions and comments that you may have.

Best wishes to you.

Kind regards,

Li Chen
