# Supplementary material for: How do public services supply, livelihood capital, and livelihood strategies affect subjective poverty?
Source: PLoS One. 2023 Oct 10;18(10):e0292651. doi: 10.1371/journal.pone.0292651 (PMC10564167; doi:10.1371/journal.pone.0292651)
Supplement: Attachment — Submitted filename: PONE-D-23-13234.docx [file pone.0292651.s003.docx]

How do public services supply, livelihood capital, and livelihood strategies affect subjective poverty?

Reviewer Comments:

Introduction:

a. The introduction provides a clear overview of the main concepts under investigation (public services supply, livelihood capital, livelihood strategies, and subjective poverty).

b. However, it would be beneficial to provide a concise definition of subjective poverty, as it may not be familiar to all readers.

c. Consider adding a brief statement on the significance or relevance of studying the relationship between these factors.

Literature Review:

a. The literature review is comprehensive and provides a solid foundation for the study.

b. It would be helpful to include more recent studies and publications, as the references primarily reflect knowledge up until September 2021.

c. Consider organizing the literature review section thematically to enhance clarity and coherence.

Research Methodology:

a. Provide a clear statement of the research objectives or research questions that will guide the study.

b. Specify the method(s) used to collect data, such as surveys, interviews, or secondary data analysis.

c. Clarify how the variables (public services supply, livelihood capital, livelihood strategies, subjective poverty) will be operationalized and measured.

d. If using quantitative methods, describe the statistical techniques that will be employed to analyze the data.

Results and Analysis:

a. As this is a reviewer comments section, no specific comments can be provided on the results and analysis section, as the information is not provided.

Discussion:

a. Consider comparing and contrasting the findings of your study with previous research to highlight similarities or differences.

b. Discuss the implications of the findings for policy and practice, addressing how public services and livelihood interventions could be improved to alleviate subjective poverty.

c. Identify any limitations of the study, such as sample size, data collection method, or potential biases.

Conclusion:

a. The conclusion should provide a concise summary of the key findings and their implications.

b. Consider providing suggestions for future research directions based on the limitations identified.

Writing Style and Clarity:

a. Proofread the manuscript for grammatical errors, typos, and inconsistencies.

b. Ensure that the writing is clear and concise, avoiding overly complex language and jargon.

c. Use appropriate headings and subheadings to enhance the organization and readability of the manuscript.

I suggest major revision for the article before acceptance
