# Supplementary material for: How do public services supply, livelihood capital, and livelihood strategies affect subjective poverty?
Source: PLoS One. 2023 Oct 10;18(10):e0292651. doi: 10.1371/journal.pone.0292651 (PMC10564167; doi:10.1371/journal.pone.0292651)
Supplement: Attachment — Submitted filename: PONE-D-23-13234R1_RR.docx [file pone.0292651.s004.docx]

**How do public services supply, livelihood capital, and livelihood strategies affect subjective poverty?**

**Review comments:**

The paper "How do public services supply, livelihood capital, and livelihood strategies affect subjective poverty?" has a few minor reviewer comments.

The introduction is well-written and gives the reader a thorough introduction to the subject. It may be enhanced, though, by giving more precise examples of how governmental services, financial resources for a livelihood, and living arrangements can impact subjective poverty.

The evaluation of the literature is thorough and includes several pertinent researches. The results of these investigations may be more critically analyzed, nevertheless, to make it better.

There includes a brief and straightforward methodology part. It might be enhanced, though, if additional information regarding the data's collection and analysis was given.

The results area is clearly laid out and simple to navigate. However, it may be enhanced by include additional explanation of the results' ramifications.

The major conclusions of the study are outlined in the conclusion, along with some suggestions for more research. It might be enhanced, though, by offering more detailed suggestions for how to enhance public services, livelihood resources, and livelihood tactics in order to lessen subjective poverty.

The paper is interesting and well-written overall. However, it may be enhanced by including more precise illustrations, critical evaluations, and discussions of the results.
