# Supplementary material for: How do public services supply, livelihood capital, and livelihood strategies affect subjective poverty?
Source: PLoS One. 2023 Oct 10;18(10):e0292651. doi: 10.1371/journal.pone.0292651 (PMC10564167; doi:10.1371/journal.pone.0292651)
Supplement: Attachment — Submitted filename: PONE-D-23-13234R2-RR.docx [file pone.0292651.s005.docx]

**The Impact Mechanism of China's Green Finance on the Transformation and Innovation of High-Energy-Consumption Enterprises**

**Reviewer comments:**

According to the criteria of the reviewers' remarks, the authors did a decent job of revising the manuscript. I advise you to accept the document after thoroughly checking it for typos.
